# Depression in Primary care: Interpersonal Counseling vs Selective serotonin reuptake inhibitors. The DEPICS Study. A multicenter randomized controlled trial. Rationale and design

**DOI:** 10.1186/1471-244X-10-97

**Published:** 2010-11-25

**Authors:** Marco Menchetti, Biancamaria Bortolotti, Paola Rucci, Paolo Scocco, Annarosa Bombi, Domenico Berardi

**Affiliations:** 1 Institute of Psychiatry, Bologna University, Bologna, Italy; 2 Department of Medicine and Public Health, Bologna, Italy; 3 Department of Psychiatry, University of Pittsburgh School of Medicine, Pittsburgh, PA, USA; 4 Psychiatric Clinic, Department of Neuroscience, Padova University, Padova, Italy; 5 Mental Health Department, ULSS 16 Padova, Padova, Italy

## Abstract

**Background:**

Depression is a frequently observed and disabling condition in primary care, mainly treated by Primary Care Physicians with antidepressant drugs. Psychological interventions are recommended as first-line treatment by the most authoritative international guidelines but few evidences are available on their efficacy and effectiveness for mild depression.

**Methods/Design:**

This multi-center randomized controlled trial was conducted in 9 Italian centres with the aim to compare the efficacy of Inter-Personal Counseling, a brief structured psychological intervention, to that of Selective Serotonin Reuptake Inhibitors. Patients with depressive symptoms referred by Primary Care Physicians to psychiatric consultation-liaison services were eligible for the study if they met the DSM-IV criteria for major depression, had a score ≥13 on the 21-item Hamilton Depression Rating Scale, and were at their first or second depressive episode. The primary outcome was remission of depressive symptoms at 2-months, defined as a HDRS score ≤ 7. Secondary outcome measures were improvement in global functioning and recurrence of depressive symptoms at 12-months. Patients who did not respond to Inter-Personal Counseling or Selective Serotonin Reuptake Inhibitors at 2-months received augmentation with the other treatment.

**Discussion:**

This trial addresses some of the shortcomings of existing trials targeting major depression in primary care by evaluating the comparative efficacy of a brief psychological intervention that could be easily disseminated, by including a sample of patients with mild/moderate depression and by using different outcome measures.

**Trial registration:**

Australian New Zealand Clinical Trials Registry ACTRN12608000479303

## Background

Major Depression (MD) is an important public health problem, associated with high levels of disability, impairment in quality of life, and increased mortality rates [1,2], similarly to severe medical conditions like congestive heart failure and diabetes [3]. Moreover, depression is associated with high health services utilization, work absenteeism, and decreased performances at work [4,5] with elevated direct and indirect social costs. Epidemiological studies showed that MD has a high prevalence in primary care ranging from 2.6% to 29.5% [6-8].

Many patients with depression seek help in primary care and an increasing proportion has been treated in this setting, especially since the availability of safe and easy to use antidepressants (ADs) [9]. However, some problems remain in the management of depression in primary care, including the insufficient duration of antidepressant treatment and the limited use of non-pharmacological options [10-12]. In particular, psychological interventions are rarely used even for those patients who could benefit from them: patients suffering from mild depression related to stressful life events and patients in which the risk/benefits ratio of antidepressants is less favourable (e.g. elderly, frail patients, patients with polypharmacotherapy, women with post-partum depression). Several reasons account for this limited use: the small number of trained therapists in primary care [13,14], the physician's positive opinion and attitude on drug treatment [15,16], the few evidence on the comparative efficacy and effectiveness of brief psychotherapies vs antidepressants.

However, psychological interventions are often preferred by primary care patients [17,18] and are recommended as first-line treatment by the most authoritative international guidelines [19,20].The APA guidelines [19] stated that antidepressants or an effective psychotherapy alone may be considered as the first-line treatment for patients with mild to moderate major depression. The National Institute for Clinical Evidence (NICE) guidelines [20] recommend not to use antidepressants routinely to treat mild depression because the risk-benefit ratio is poor and suggest as an alternative a range of low-intensity psychosocial interventions.

These recommendations are based on few trials directly comparing antidepressant drugs with different kinds of psychological interventions in primary care [21-28]. Therefore the NICE guidelines underscore that adequately powered Randomized Controlled Trial (RCT) are warranted with representative participant samples, reporting relevant outcomes to assess the efficacy of psychological interventions and antidepressants [20].

The objectives of the RCT described in this paper are: 1) to evaluate the efficacy of a psychological intervention, the Inter-Personal Counseling (IPC), compared with Selective Serotonin Reuptake Inhibitors (SSRIs), for mild to moderate MD; 2) and to assess the efficacy of treatment augmentation with SSRI or IPC in patients who do not respond to monotherapy.

## Methods/Design

### Study procedures

#### Study design

This multi-center randomized controlled trial was conducted in nine academic centres located in Northern, Central and Southern Italy: Bologna (coordinating centre), Bari, Cagliari, Foggia, Modena, Pavia, Perugia, Torino, Varese. The recruitment phase started on May 1st 2006 and finished on May 1st 2008. Each centre set up a specific collaborative programme with primary care physicians working in its area, with the aim to improve the management of depression. In some research units a collaborative programme was already in place at study inception, in others it was implemented on the occasion of the research. Primary Care Physicians (PCPs) were informed about the DEPICS study protocol during specific meetings and informal contacts; further, written materials about eligibility criteria and treatment algorithm were delivered.

Patients were recruited from university-based psychiatric consultation-liaison services specifically dedicated to PCPs. PCPs were encouraged to refer patients recognized as suffering from depressive symptoms; patients were seen by a consultant psychiatrist and evaluated for the possible inclusion in the study.

#### Study protocol approval and trial registration

Participation in the study was voluntary and written informed consent was obtained. Patients were informed that they could withdraw their consent to participate at any time, with no negative consequences on their future medical treatment. Patients who wished to withdraw from the study received care as usual. The study protocol was approved by the Ethical Committee of Azienda Ospedaliera Universitaria di Bologna. Eligible patients received an explanation of the study procedures and were asked to sign an informed consent. Written documentation about study methodology and procedures and a letter for PCP were delivered to all recruited patients. The PCPs were also periodically informed by letter or by phone about the course of illness of their patients during the study.

The trial was registered in the Australian New Zealand Clinical Trials Registry (ANZCTR) as ACTRN12608000479303.

#### Inclusion and exclusion criteria

Adult patients suffering from depressive symptoms and referred from their PCP were potential candidates for the trial. Patients aged 18 years or older were eligible if they met criteria for a MD Episode, had a score ≥13 on Hamilton Depression Rating Scale (HDRS, 21-item version) [29], and were at their first or second MD Episode treated with antidepressants or psychotherapy. Patients who met all inclusion/exclusion criteria but had a HDRS score < 13 were reassessed after 1 month (watchful waiting arm); if their HDRS score was ≥13 after 1 month they were enrolled and randomised to IPC or SSRI. Inclusion and exclusion criteria are shown in Table 1.

**Table 1:** Study recruitment: inclusion and exclusion criteria

Inclusion criteria	Exclusion criteria
Age ≥18 years	Current moderate to high suicide risk
DSM-IV Major Depressive Episode (MDE)	Current/past episodes of mania or hypomania
HDRS score ≥13	Current/past psychotic symptoms
First or second lifetime treated MDE°	Borderline personality disorders
Availability to sign informed consent	Anti-social personality disorders
	Substance abuse or dependence
	Cognitive impairment
	Effective ongoing antidepressant treatment*
	Effective ongoing psychotherapy
	Poor knowledge of Italian language
	Pregnancy or breastfeeding

° Only previous episodes treated with specific therapeutic options (antidepressants, psychotherapy) were considered.

* Including antidepressants, mood stabilizers, and antipsychotics; only use of moderate dose of sedatives-hypnotics was allowed (doses ≤ 7.5 mg lorazepam equivalent/day).

We chose to exclude patients with two or more previous depressive episodes treated with antidepressants or psychotherapy, with Borderline or Antisocial Personality Disorder because of the different pattern of response to treatment and the less favourable prognosis [30,31].

After a baseline assessment, eligible patients were randomly allocated to a brief structured psychological intervention, IPC, or to antidepressant treatment with SSRI, the most used class of drugs in primary care.

#### Randomization and treatment allocation

Randomization sequences, derived from a computer random number generator, were delivered to each centre by the coordinating centre. In each centre, allocation to treatment group was made by dedicated research personnel outside the consultation-liaison service where patients were recruited, assessed and treated. After baseline assessment and after consent to participation in the study was obtained, the researcher was contacted via telephone by clinicians and disclosed the assignment.

#### Blinding

Raters who administered assessment's instruments were different from the clinicians who provided psychiatric consultation to PCPs and delivered pharmacological or psychological interventions. Efforts were made to keep raters blind to randomization assignment.

#### Outcomes

**Figure 1:**
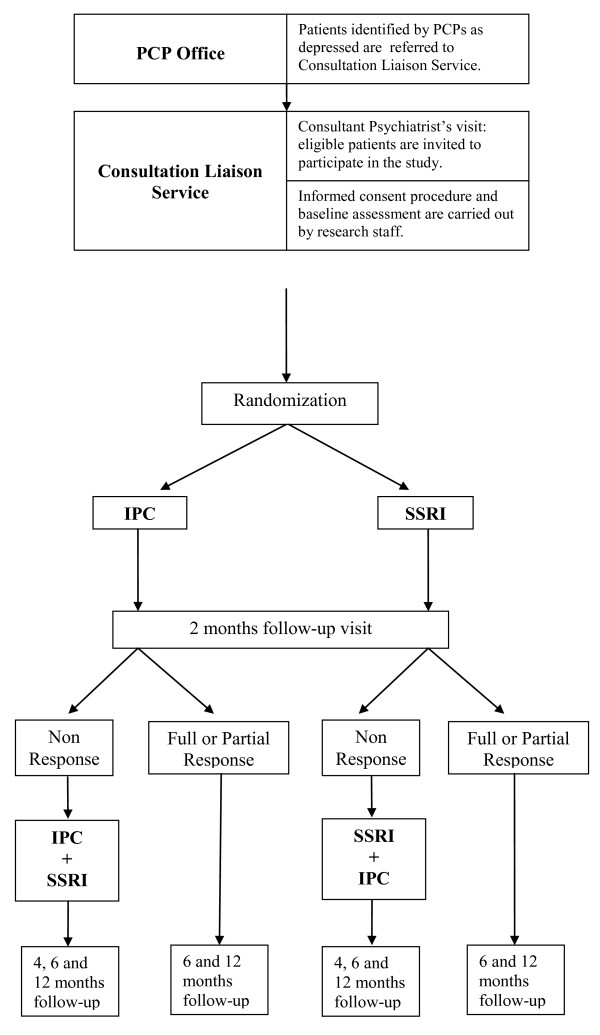
**Contains study design**.

The primary outcome was the remission of depressive symptoms at the 2-month follow up visit, defined as a HDRS score of 7 or less. Secondary outcome measures were: improvement in global functioning and recurrence of depressive symptoms at the 12-months follow up visit. Patients with a HDRS score ≥ 13 at 2-month follow-up received augmentation with other treatment (Figure 1).

### Intervention

#### Interpersonal Counseling (IPC)

IPC is a brief manualized psychological intervention, derived from Interpersonal Psychotherapy (IPT) and suitable for different medical contexts. This intervention can be delivered by trained mental health professionals, nurses and social workers [32-35]. IPC has been adapted and tested on patients with several conditions, in association with pharmacological treatment or alone. These conditions include subthreshold depression [36], major depression [37], depressive symptomatology after miscarriage [38], psychological symptoms after a major physical trauma [39], stress and distress without serious medical or psychiatric conditions [32], breast cancer [40], and distress after myocardial infarction [41].

IPC is focused on patients' current psychological problems and social functioning and specifically on four interpersonal problem areas: prolonged grief, interpersonal disputes, role transitions and interpersonal deficits. Patients are helped to identify effective strategies in order to deal with their interpersonal problems. In the original manual [33], IPC consisted of six thirty-minute sessions, with the initial session being longer (1 hour), and was self-dosing: the patient determined the number of sessions and many patients were satisfied with fewer than six sessions. In our study IPC has been adapted to accommodate patients' needs. The recommended number of therapy sessions was six thirty-minute weekly sessions. Therapists determined if one or two additional sessions were needed (6+1 or 6+2). IPC sessions were videotaped if patients provided a written informed consent.

##### Training of IPC therapists

IPC was delivered by 18 therapists (Bologna 3, Modena 2, Torino 3, Pavia 2, Varese 2, Perugia 1, Foggia 3, Bari 1, Cagliari 1). Therapists were residents in psychiatry or in clinical psychology with a clinical experience of at least 2 years. They attended a 3-day teaching seminar on interpersonal theory's foundations and IPC structure and techniques conducted by one of the authors (PS), who had previously trained and supervised the Italian IP-therapists of a cross-national RCT [42,43]. Before starting the formal training, trainees were asked to read the Italian translation of the IPT manual [44] and the IPC treatment manual by Weissman [30]. During the teaching seminars, videotapes of psychotherapy sessions were presented and discussed, before simulating an IPC session with a role-playing. The candidates were encouraged to use the interpersonal approach when treating their patients (out of the trial).

##### IPC group supervision

Subsequently, monthly group supervisions were scheduled with one of the authors (PS) in order to discuss cases, to ensure the intervention's consistency. At each meeting the trainees reported the problems and challenges they experienced with IPC. Trainees were requested to present four or five IPC videotaped sessions to review during group supervision, choosing the more challenging sessions. These videotapes provided indications on the communication style, techniques and strategies applied by therapists. In this way, during the supervision meeting, trainees could learn from the supervisor, but also vicariously from one another (peer-supervision). Working with a group of trainees means that each could be learning even while one is the particular focus of the supervisor attention. The supervisor stimulated trainees to use a peer-supervision method with all the participants actively involved in the case discussion with suggestions or comments. As Hillerbrand [45] pointed out, group members are often able to give feedback to one another that is more understandable than what the supervisor offers. Then, peer-supervision was used in any unit between the monthly-group supervisions.

### Antidepressant treatment

Because our aim was to assess the antidepressant treatment for depression as delivered routinely in clinical practice, we chose two antidepressants, sertraline and citalopram, among the available SSRIs. These agents have minimal or modest effects on the cytochrome P-450 isoenzyme system which makes them safe even in patients with physical illnesses and taking other medications. Moreover, sertraline and citalopram had the lowest cost among SSRI antidepressants in Italy at study start.

Citalopram was started at 10-20 mg and titrated if needed to 60 mg and sertraline was started at 25-50 mg and titrated up to 200 mg. The pharmacological treatment was continued for at least 4-6 months after the patient had responded as suggested by international guidelines [19,20]. The psychiatrist was free to choose between the two pharmacological options according to his/her personal preference and informed the PCP by letter about the suggested pharmacological treatment. Moreover he/she provided psycho-education about antidepressants and their side effects. Two or three subsequent visits with the consultant psychiatrist were planned every 2-3 weeks, lasting around 15 minutes in order to evaluate patients' compliance, clinical response and side effects; a specific form was used to assess adverse events. The only treatment modification allowed was the switch from sertraline to citalopram and vice versa.

There were no limitations on the number of PCP's visits. Concurrent use of sedatives-hypnotics was allowed up to a dose < 7.5 mg lorazepam equivalent/day in both treatment arms. During the study, other psychotropic medications were forbidden; if needed, the patient was terminated from the protocol and referred to his or her PCP for alternative treatments.

### Combined treatment

Patients with a HDRS score ≥13 at 2-months follow-up received augmentation with other treatment. In particular those in the SSRI arm began a regular 6-sessions IPC; those in IPC arm began the antidepressant therapy and continued IPC for further 4-6 sessions.

### Assessments

At baseline, patients filled out socio-demographic and clinical forms, including information about medical history, current pharmacological treatments and health services utilization in the last 6 months (including information about use of both general health and specialist care and about laboratory tests, instrumental procedures, admission to hospital). Follow-up visits were scheduled at 2, 4, 6 months and 1 year; the 4-month assessment was scheduled only for non-responders who started the combined treatment at 2 months. Table 2 summarizes instruments administered at each visits.

**Table 2:** Assessment instruments at each time point

Instrument	Baseline		Follow-up visits		
		**2 months**	**4 months**	**6 months**	**12 months**

MINI Plus	X				X
SCID II	X				
HDRS-21	X	X	X	X	X
GDS (only patients aged 65 or more)	X	X	X	X	X
WSAS	X	X	X	X	X
WHOQOL BREF	X	X	X	X	X
IIP-64	X				X
IPQ	X	X			X
ASQ	X				
GSQ		X		X	X

List of abbreviations: ASQ = Attachment Style Questionnaire, GDS = Geriatric Depression Scale, GSQ = General Satisfaction Questionnaire, HDRS = Hamilton Depression Rating Scale, IPQ = Interpersonal Problem Questionnaire, IIP-64 = 64-item Inventory of Interpersonal Problems, MINI Plus = Mini International Neuropsychiatry Interview Plus, SCID II = Structured Clinical Interview for DSM-IV Axis II Personality Disorders, WSAS = Work and Social Adjustment Scale, WHOQOL = World Health Organization Quality Of Life.

The assessment was conducted by interviewers not involved in patients treatment and trained to the use of instruments and scales. Research personnel was trained to the use of study instruments in 2 investigator's meetings, in which videotaped interviews were presented and rated. In particular, a structured interview guide for the HDRS was employed in order to facilitate the achievement of good inter-rater reliability [46]. Certification in the use of the HDRS was obtained when the total score did not differ by more than 4 points with the gold standard (MM) on 3 videotaped cases.

### Diagnostic assessment

The diagnostic assessment was carried out at baseline using the Mini International Neuropsychiatry Interview (MINI) Plus [47], an instrument that allows to make a diagnosis according to DSM-IV or International Classification of Diseases (ICD-10) criteria. The reliability, sensitivity, and specificity of the MINI are equivalent to those of the Composite International Diagnostic Interview (CIDI) [48] and the Structured Clinical Interview for DSM-IV [47] in a clinical population.

The assessment was completed with the section on diagnosis of borderline personality disorders of the SCID-II; we used the self-report scale followed, if necessary, by a semi-structured interview [49].

### Depressive symptoms

Severity of depressive symptoms was assessed using the 21-item Hamilton Rating Scale for Depression (HDRS-21) [29] the most widely used outcome measure in trials on depression. A HDRS score < = 7 denotes the absence of depression, from 8 to 17 mild depression, from 18 to 24 moderate depression and a score ≥25 indicates severe depression [50]. In patients aged 65 years or more depressive symptom severity was also evaluated using the Geriatric Depression Scale (GDS) [51].

### Functioning

We used the Work and Social Adjustment Scale (WSAS), a self-report 5-item scale that measures functional impairment attributable to an identified problem or disorder. WSAS items investigate ability to work, home management, social leisure, private leisure, and relationships. A score from 11 to 20 denotes mild disability, while a score higher than 20 denotes severe disability [52].

### Quality of life

The World Health Organization Quality Of Life (WHOQOL) - BREF is used to assess quality of life [53]. It consists of 24 items organized into four domains: physical health, psychological health, social relationships, and environment. Two additional items measure the overall perception of quality of life and health. Domain scores are scaled in a positive direction: higher scores denote higher quality of life [54].

### Interpersonal areas

We used two instruments:

1) Interpersonal Problems Questionnaire (IPQ) is a brief self-report assessment measure, which queries life events that are relevant to the four focal interpersonal problem areas addressed in IPT: unresolved grief, role transitions, interpersonal role disputes and interpersonal deficits. [55]. It includes 3 different sections, the first investigating interpersonal relationships, the second focused on social life and close relationships and the last exploring the presence of significant life events occurred in the past 12 months.

2) The 64-item Inventory of Interpersonal Problems (IIP-64) is self-report measure of maladaptive relationship behaviour used to identify dysfunctional patterns in interpersonal interactions. This inventory assesses the severity of interpersonal problems in 8 domains: domineering or controlling, vindictive or self-centred, cold and distant, socially inhibited, non-assertive, overly accommodating, self-sacrificing, and intrusive or needy. The IIP-64 has been widely used to assess psychotherapy [56].

### Other instruments used in the study

In patients aged 65 years or more, if cognitive impairment was suspected, the Mini Mental State Examination (MMSE) was administered at baseline [57]; patients with a score of 27 or less were excluded from the study.

Moreover, we used the Attachment Style Questionnaire (ASQ), a 40-item self-report Likert Scale specifically designed to assess the type of attachment to significant other. The ASQ includes five scales: confidence (in self and others), discomfort with closeness, need for approval, preoccupation with relationships, and relationship as secondary (to achievement). Each sub-scale represents a dimension central to adult attachment and each item is scored on a 6-point scale ranging from totally agree to totally disagree [58].

Patient's satisfaction was measured only at the follow up visits with the General Satisfaction Questionnaire (GSQ), a self-report 7-item assessment scale that measures satisfaction with treatment received and with health services accessibility and acceptability [59].

### Data analysis

#### Data management and study monitoring

Data were recorded by research assistants in a database developed in MS-ACCESS. The data quality was checked on a regular basis to ensure that data analysis could be promptly conducted.

#### Sample size

The sample size was determined using remission measured with the HDRS as the primary outcome. Data from previous RCT on MD showed that the remission rate with SSRI treatment is 35%[60]. Other studies conducted in primary care reported higher remission rates with SSRI, ranging from 52 to 67% [24,61]. Assuming an intermediate 45% remission rate with SSRI and a clinically significant difference of 15% between the two treatments, a sample size of 274 (137 per arm) was determined that would result in a power of 80% at 0.05 alpha level. Considering a drop-out rate of about 10% at the first 2 months follow up, we planned to enrol 300 patients (150 per arm).

### Statistical analysis

The statistical analysis plan includes the use of logistic regression analysis to model the probability of remission at 2 and 6 months as a function of randomization assignment. For this analysis, patients dropped out from the study are considered as non-remitters. The same model is used to predict the need of combined treatment (coded as yes/no) as a function of patient demographic and clinical characteristics.

Linear mixed models are used to analyse functional change and change in quality of life from baseline as a function of treatment, using the baseline score as a covariate. Satisfaction with treatment is compared between the treatment arms using Mann-Whitney test.

## Discussion

Many studies have been conducted on the treatment of depression, but mostly on patients with moderate to severe forms in the mental health setting. Relatively few trials have been carried out with patients with mild depression in primary care. In particular, evidence on the efficacy of psychological interventions in comparison with antidepressants is scanty. To our knowledge only eight RCTs comparing different psychological interventions and antidepressants were conducted in primary care [21-28]. However, methodological issues limit the findings of these trials: first,, they had very small sample sizes, thereby increasing the likelihood of Type II error; moreover the three older ones used tricyclic antidepressants that are now rarely prescribed [21-23]; finally, two studies recruited only patients suffering from minor depression or dysthimia [27,28]. As suggested by several authors, it is crucial that RCTs on psychological interventions conducted in primary care include representative participant samples, use multiple outcome measures, test interventions easy to disseminate and evaluate differences in treatment response as a function of co-existent psychiatric problems and other medical problems [13,62-64]. Considering the challenges researchers must overcome, the DEPICS study has some points of strengths:

1) The use of broad eligibility criteria in order to obtain a representative sample, including patients with mild depression and depressive disorders comorbid with anxiety disorders or physical illnesses, very common in primary care setting.

2) The use of different outcome measures and a broad assessment including severity of depressive symptom, functioning, quality of life, interpersonal relationships, and patient's satisfaction,

3) The evaluation of the efficacy of a brief structured psychological intervention (IPC) as a monotherapy for MD in primary care.

Among evidence-based psychotherapies for depression, the interpersonal approach is one of the most supported, but qualified psychotherapists are often not available in primary care. If we want to see widespread implementation of effective practices such as evidence -based psychotherapies, we need to develop and test "simpler" versions of evidence based psychosocial treatment that could be provided by different health-care professionals who do not necessarily have mental health training [64]. With a view of moving from research evidence to practice, IPC lends itself to be disseminated in primary care, because it is a briefer version of evidence-based psychosocial treatments and could be provided by clinicians with less mental health expertise than IPT or CBT therapists. The efficacy of IPC has been previously tested in patients with depressive symptoms related to current stress (mainly physical illnesses) and subthreshold depression or in individual, group and telephone format [32,37-41,65]. One study evaluated the efficacy of IPC plus venlafaxine for MD in primary care [36]. Moreover, Bolton et al [65] tested the efficacy of group IPC in alleviating depressive symptoms and dysfunction in patients with sub-threshold or major depression in Uganda. However, IPC was not previously tested as an individual intervention in monotherapy for patients with MD in primary care.

An important limitation of this study is the absence of a placebo group, usually characterized by a high rate of spontaneous remission (27% to 43%) [22,66,67]. However, the present study is part of a more comprehensive collaborative programme between primary care and mental health services and we chose to employ only an active comparator in the protocol to match the real-world clinical setting as closely as possible. Another limitation is the exclusion of patients with more than one clinically significant depressive episode in their personal history. We chose to exclude these patients because of the different pattern of response to treatment and the less favourable prognosis [9]. Our results therefore cannot be generalized to patients with chronic or recurrent MD.

In conclusion, the present trial aims to provide evidence on the efficacy of a brief and easy to implement psychological intervention compared to the most frequently used drug treatments for major depression.

## Competing interests

The authors declare that they have no competing interests.

## Authors' contributions

DB, MM, BB conceived the study and developed the study protocol. DB is the principal investigator that assembled the group of investigators. MM and BB wrote the first draft of this manuscript, describing the trial protocol. PR provided statistical advice in the design of the study and its on-going evolution. PS, MM and BB participated in the design and in the planning of the interventions. PR, BB and AB managed the trial data. All authors have read and corrected draft versions, and approved the final version.

## Pre-publication history

The pre-publication history for this paper can be accessed here:

http://www.biomedcentral.com/1471-244X/10/97/prepub
